# Interactions between Blood-Borne *Streptococcus pneumoniae* and the Blood-Brain Barrier Preceding Meningitis

**DOI:** 10.1371/journal.pone.0068408

**Published:** 2013-07-16

**Authors:** Federico Iovino, Carlos J. Orihuela, Henk E. Moorlag, Grietje Molema, Jetta J. E. Bijlsma

**Affiliations:** 1 Department of Medical Microbiology, University Medical Center Groningen, University of Groningen, Groningen, The Netherlands; 2 Department of Microbiology and Immunology, University of Texas Health Science Center at San Antonio, San Antonio, Texas, United States of America; 3 Department of Pathology and Medical Biology, Medical Biology Section, University Medical Center Groningen, University of Groningen, Groningen, The Netherlands; University of Padova, Medical School, Italy

## Abstract

*Streptococcus pneumoniae* (the pneumococcus) is a Gram-positive bacterium and the predominant cause of bacterial meningitis. Meningitis is thought to occur as the result of pneumococci crossing the blood-brain barrier to invade the Central Nervous System (CNS); yet little is known about the steps preceding immediate disease development. To study the interactions between pneumococci and the vascular endothelium of the blood-brain barrier prior to meningitis we used an established bacteremia-derived meningitis model in combination with immunofluorescent imaging. Brain tissue of mice infected with *S. pneumoniae* strain TIGR4, a clinical meningitis isolate, was investigated for the location of the bacteria in relation to the brain vasculature in various compartments. We observed that *S. pneumoniae* adhered preferentially to the subarachnoid vessels, and subsequently, over time, reached the more internal cerebral areas including the cerebral cortex, septum, and choroid plexus. Interestingly, pneumococci were not detected in the choroid plexus till 8 hours-post infection. In contrast to the lungs, little to no leukocyte recruitment to the brain was observed over time, though Iba-1 and GFAP staining showed that microglia and astrocytes were activated as soon as 1 hour post-infection. Our results imply that i) the local immune system of the brain is activated immediately upon entry of bacteria into the bloodstream and that ii) adhesion to the blood brain barrier is spatiotemporally controlled at different sites throughout the brain. These results provide new information on these two important steps towards the development of pneumococcal meningitis.

## Introduction

*Streptococcus pneumoniae* (the pneumococcus) is a Gram-positive human pathogen that causes life-threatening invasive diseases such as pneumonia and bacteremia with high morbidity and mortality throughout the world. Moreover, *S. pneumoniae* is now the most common etiological agent of bacterial meningitis in the United States and Europe [1]. Meningitis is an infection of the Central Nervous System (CNS), which is characterized by inflammation of the protective membranes covering the brain and spinal cord, collectively known as the meninges [2], [3]. One entry route for *S. pneumoniae* into the CNS is thought to be via the bloodstream by crossing the blood vessels of the blood-brain barrier. The blood-brain barrier is composed of a specialized vasculature covered by endothelial cells that protects the brain from harmful substances that are present in the blood stream and supplies the brain with the required nutrients for its proper functions, making the brain an immune privileged organ [2], [3]. The entry of pathogens into the CNS leads to inflammation, local production of pro-inflammatory cytokines and activation of the endothelium. The blood-brain barrier becomes more permeable allowing for the influx of large numbers of leukocytes into the brain. This influx of leukocytes during the inflammatory response leads to intracerebral edema and swelling of the meninges and these pathological changes lead to neurological damage and in some cases death of the patient [1], [3]. High levels of the pro-inflammatory cytokines such as interleukin 1 beta (IL-1 beta), interleukin 6 (IL-6) and tumor necrosis factor alpha (TNF alpha), were detected in the cerebro-spinal fluid (CSF) of patients with meningitis, which is typical of the severe inflammation of the brain [4]. Astrocytes, also known as astroglia, are characteristic star-shaped cells in the brain. They are the most abundant cells in the human brain and they have several important functions, including biochemical support for endothelial cells of the blood-brain barrier, provision of nutrients to the nervous tissue, and repair of the brain and spinal cord following traumatic injuries [5], [6]. Microglia are the resident macrophages of the brain and spinal cord and they act as first immune defense of the CNS in response to infections [6], [7]. Microglia are able to phagocytose foreign molecules and present them to T cells. Phagocytic microglia travel to the site of injury and release pro-inflammatory factors to promote the proliferation of more microglial cells. Microglia also interact with astrocytes and neurons to fight and eradicate the infections as quickly as possible [6], [7], [8]. During inflammation microglia and astrocytes get activated and their cellular morphology undergoes dramatic changes in a process called astrogliosis for astrocytes: the soma becomes more round and the dentrites shorter and thicker [9], [10], [11], [12].

How bacterial pathogens cross the blood-brain barrier is currently unclear. Several studies have implicated 1) the destruction of the endothelial cell layers by *N. meningitidis* and pneumococcal pneumolysin [13], [14], 2) traversal of the blood-brain barrier in between the cells by disruption of the tight junctions [15], and 3) bacterial traversal of the blood-brain barrier by transcytosis, an intracellular transport route designed to transport molecules and vesicles through cells from the apical to basolateral side [16].

The blood circulatory system of the brain comprises the microvasculature, which includes arterioles, capillaries, and venules, and the macrovasculature, containing arteries and veins [17]. At present, it is unknown whether *S. pneumoniae* interacts with both the micro- and the macrovasculature prior to invasion into the brain, when it interacts with the vasculature in different areas in the brain, and whether all endothelial cells along the microvascular tree engage in bacterial adhesion and transmigration. It is thought that an important site of entry might be the choroid plexus [18], as shown for *Streptococcus suis*, which induces blood-brain barrier disruption in porcine choroid plexus [19], and may also translocate intracellular across the choroid plexus epithelium [20]. In human brain microvascular endothelial cells (HBMEC), pneumococci were able to adhere to the platelet-activating factor (PAF) receptor expressed by these cells, allowing transmigration through the endothelial cell to the basolateral site [16], [21]. Concordantly, PAF receptor deficient mice showed a decreased incidence of pneumococcal meningitis after intravenous challenge due to less pneumococcal translocation across the blood-brain barrier [21]. *In vitro* and animal experiments showed in strain D39 that the pneumococcal surface protein C PspC (also known as CbpA) are required for development of bacterial meningitis, and that TIGR4 derived recombinant PspC binds to laminin receptor; which is also targeted by other neurotropic pathogens including prions and bacteria such as *Haemophilus influenzae* and *Neisseria meningitidis* [22].

Summarizing, while pneumococcal translocation occurs during natural disease pathogenesis, it is still unclear how and where this process takes place *in vivo*. The aim of this study was to investigate the interactions between *S. pneumoniae* and the brain endothelium before the development of meningitis. Since an important route of CNS infection by bacterial pathogens is via the blood stream, we challenged mice with *S. pneumoniae* through intravenous injection. Herein we report that, *S. pneumoniae* indeed adheres tightly to the vascular endothelium, preferentially to the subarachnoid vessels and over time, increasingly to the endothelium of the cerebral cortex, septum and choroid plexus. This adhesion results in almost immediate microglial and astroglial activation but does not lead to leukocyte recruitment. Thus, our studies suggest that *S. pneumoniae* CNS invasion is spatiotemporal separated.

## Materials and Methods

### Bacterial Strains and Growth Conditions

An encapsulated *S. pneumoniae,* serotype 4 strain, TIGR4 [22], was used in this study. Pneumococci were grown standing in Todd-Hewitt broth (Oxoid Thermo Scientific, Basingstoke, England) at 37°C, bacterial growth was monitored by measuring the optical density (OD) at 600 nm with a spectrophotometer. Bacteria were harvested at OD_600_ = 0.25–0.30 by centrifugation of 1 ml of culture at 10000 rpm for 3 minutes, and the bacteria were suspended in 1 ml of sterile phosphate buffered saline (PBS) (Lonza, Verviers, Belgium). Serial dilutions in sterile PBS were made and plated on blood-agar plates to calculate the dilutions for the challenge dose of 10^7^ colony forming unit (CFU) for each mouse.

### Bacteremia Derived Meningitis Model

All experiments involving animals were performed with the prior approval of and in accordance with guidelines of the Institutional Animal Care and Use Committee of the University of Groningen (DEC nr. 6152A). The bacteremia derived meningitis model described by Orihuela et al. [22] was performed in the following way: four groups of 5 female Balb/c mice, 6 to 8 weeks old (Harlan, Horst, NL) were anesthetized by inhalation of 2.5% isofluorane before the challenge. Intravenous tail vein injection with 200 µl of 10^7^ CFU of the TIGR4 wild type was performed, as control mice were injected with PBS (mock-infected). The mice were sacrificed at 1, 3, 8, and 14 hours after bacterial challenge, the mock-infected mice after 3 hours. After sacrifice, to remove unattached bacteria in the blood stream, perfusion was performed by injecting sterile PBS in the right ventricle via the vena cava until the blood was completely removed. Brains, lungs, and spleens were collected and stored with Shandon Cryomatrix (Thermo Scientific, Runcorn, ENG) at −80°C.

### Cell Lines and Culture Conditions

Human Brain Microvascular Endothelial Cells (HBMEC) [23] (a kind gift from Dr. K.S. Kim) were cultivated in RPMI-1640 (Biochrom, Berlin, GER) supplemented with 10% Fetal Calf Serum (FCS) (Biochrom), 10% Nu-serum (BD Biosciences, Breda, NL), 2 mM L-glutamine (Gibco, Grand Island, United States), 1 mM sodium pyruvate (Gibco), 1% MEM-vitamines (Gibco) and 1% non-essential amino acids (Gibco). HBMEC were split in T25 flasks (TPP, Trasadingen, Switzerland) after reaching confluency and cultured till passage 36.

### Pneumococcal Adherence to Endothelial Cells

HBMEC were grown on glass disks (Thermo Scientific, Braunschweig, Germany) placed inside wells of 12 well-plates (TPP, Trasadingen, Switzerland). Cells were grown to confluency at 37°C with 5% CO_2_. After washing the cells with sterile PBS, 900 µl cell culture medium was added to each well and 100 µl containing approximately 10^6^ CFU of *S. pneumoniae* TIGR4 was added. After 1 hour incubation at 37°C/5% CO_2_ cells were washed 3 times with PBS and fixed with paraformaldehyde (Sigma Aldrich) 4% solution in PBS.

### Antibodies, Lectin and Isotype Controls

The following antibody combinations and dilutions were used for immunofluorescent detection, all dilutions were made in sterile PBS with 5% Fetal Calf Serum (FCS) (Biochrom, Berlin, GER). To detect *S. pneumoniae,* an anti capsule serotype 4 antibody (Statens Serum Institute, Copenhagen, DEN) 1∶200 diluted was used, followed by Alexa Fluor 488 goat anti-rabbit (Invitrogen Life Technologies, Carlsbad, CA) 1∶500 diluted. As control for the bacteria detection, brain sections from mock treated mice were incubated with anti capsule serotype 4 antibody, followed by Alexa Fluor 488 goat anti rabbit, and no pneumococci were detected (Figure S1). For the detection of endothelial cells, DyLight 594 labeled LEL (Vector Laboratories, Burlingame, CA) was used in a 1∶200 dilution. LEL binds well to glycophorin and Tamm-Horsfall glycoprotein and has been used effectively to label vascular endothelium in rodents (http://www.vectorlabs.com/catalog.aspx?prodID=1715). For the detection of VE-Cadherin in mouse tissue, a rat anti mouse VE-Cadherin antibody 1∶50 diluted (kind gift from Dr. E. Dejana, FIRC Institute of Molecular Oncology, Milan, Italy) was used, while for the detection of VE-Cadherin in HBMEC a mouse anti human VE-Cadherin antibody (Cell Signaling, Danvers, United States) 1∶400 diluted was used, respectively followed by incubation with Alexa Fluor 594 goat anti rat and Alexa Fluor 594 goat anti mouse both 1∶500 diluted. As leukocyte marker, CD45 antibody 1∶50 diluted (Dako, Ely, UK) was used followed by Alexa Fluor 488 goat anti-rat antibody (Invitrogen Life Technologies). For the detection of astrocytes, an anti-Glial Fibrillary Acidic Protein (GFAP) antibody (Dako) at 1∶400 dilution was used, followed by Alexa Fluor 488 goat anti rabbit antibody (Invitrogen Life Technologies). Microglia were detected with goat anti-mouse ionized calcium-binding adapter molecule 1 (Iba-1) (Abcam, Cambridge, United Kingdom) followed by Alexa Fluor 488 donkey anti-goat antibody (Invitrogen Life Technologies). As isotype controls for the primary antibodies the following antibodies were used at the same dilution as those for specific primary antibodies: an anti avi-Tag antibody (GenScript, Piscataway, NJ; rabbit IgG isotype control), anti-human CD4 antibody (AbD Serotec, Martinsried, Germany; rat IgG isotype control), mouse IgG (Innovative Research, Plymouth, United States), goat IgG (Santa Cruz Biotechnology, Heidelberg, Germany). The avi-Tag antibody was used in combination with Alexa Fluor 488 goat anti-rabbit antibody, the anti-CD4 antibody was combined with Alexa Fluor 488 goat anti-rat and the mouse IgG was combined with Alexa Fluor 594 goat anti-mouse. No fluorescence was detected in these controls (data not shown). Incubation of the tissue sections with only the secondary antibodies did not result in a fluorescence signal either. For the immunohistochemical detection of CD31, rat anti mouse CD31 antibody (Pharmigen BD Biosciences, San Jose, CA) at a 1∶50 dilution was used, conjugated with secondary antibody rabbit anti- rat IgG 1∶40 diluted. As isotype control a rat anti human CD4 antibody at the same dilution of the rat anti-mouse CD31 antibody was used.

### Immunofluorescent Detection

Brain sections of 5 µm thin were cut with a cryostat and placed on microscope glass slides (3 sections/slide) (Starfrost, Dallas, TX) and dried under a fan for at least 30 minutes for a better attachment of the section on the glass slide. Sections were fixed with acetone for 10 minutes, dried and next incubated with primary antibody for 60 minutes. Slides were washed in PBS 3 times for 5 minutes. Subsequently, each section was incubated with a mixture of the appropriate secondary antibodies (see *Antibodies, lectin and isotype controls*) and DyLight 594 labeled LEL for 45 minutes in the dark. After washing with PBS 3 times for 5 minutes, the slides were incubated in the dark with DAPI (Roche, Mannheim, Germany) 1∶5000 diluted. After washing again twice for 5 minutes in PBS, Citifluor solution (Science Services, Munich, Germany) was added to each section after which the coverslip was applied.

For GFAP and Iba-1 staining, brain sections of 30 µm thickness were fixed with 4% paraformaldehyde in PBS for 20 minutes. The slides were washed in PBS 3 times for 5 minutes and preincubated using PBS with 0.3% Triton X-100 (Sigma Aldrich) and 2% BSA (Sigma Aldrich) for 30 minutes. Each tissue section was incubated overnight at 4°C with primary antibody diluted in PBS with 0.3% Triton X-100 and 1% BSA. The slides were washed three times with PBS for 5 minutes. Each tissue section was incubated with the secondary antibody diluted in PBS with 0.3% Triton X-100 and 1% BSA.

For immunohistochemical staining of CD31, brain sections were cryostat cut at 5 µm, mounted onto glass slides (Starfrost) and fixed with acetone for 10 minutes. After drying, sections were incubated for 45 minutes at room temperature with primary rat anti-mouse CD31 antibody in the presence of 5% FCS. After washing, endogenous peroxidase was blocked by incubation with 0.1% H_2_O_2_ in PBS for 20 minutes. This was followed by incubation for 30 minutes at room temperature with horseradish peroxidase (HRP) conjugated secondary antibody rabbit anti rat IgG (Dako) 1∶40 diluted. Conjugate was diluted 1∶50 in PBS supplemented with 2% normal mouse serum. Between incubation with antibodies, sections were washed extensively with PBS. Peroxidase activity was detected with 3-amino-9-ethylcarbazole (AEC) (Sigma Aldrich, St. Louis, USA) and sections were counterstained with Mayer’s hematoxylin (Klinipath, Duiven, NED).

For the VE-Cadherin staining, HBMEC and HUVEC were incubated with a mixture of anti VE-Cadherin antibody and anti capsule serotype 4 antibody after fixation. Afterwards, cells were washed 2 times with PBS and incubated with a mixture of proper secondary antibodies. Cells were washed again 2 times with PBS.

Citifluor solution (Science Services, Munich, Germany) was added to each tissue section/glass disk after which the coverslip was applied. The slides were analyzed with a Leica DM5500B microscope, using “Fluorescence” or “Contrast Phase” mode depending on immunofluorescent or immunohistochemical staining. Images were taken with a Leica DFC 360 FX camera. Additionally a Leica SP2 AOBS confocal microscope was used to confirm the presence of adhered pneumococci to the brain vascular endothelium.

### Image Processing

All the images obtained with fluorescence microscope Leica DM5500B were processed by Image J (http://rsb.info.nih.gov/ij/) [24]. The TIFF images obtained with the 350 nm (blue), 488 nm (green) and 594 nm (red) wavelength filters were merged using the *Color-Merge Channels* function. As minor manipulation, background correction was applied in those images where the background was relatively or even across the image by using the *Brightness & Contrast* command of Image J. In all the images subjected to this minor adjustment, the background subtraction was applied to all parts of the image. The images of the immunohistochemical staining of CD31 were taken in grayscale by the Leica QWin Standard software. The LEI z-stacks obtained with the confocal microscope Leica SP2 AOBS were merged through Imaris (Bitplane Scientific Software). No digital adjustment was performed on these images. Imaris was used also to generate that XZ and YZ plane orthogonal views.

### RNA Isolation

Frozen murine brains were mixed with 0.5 ml TRIzol (Invitrogen Life Technologies) under liquid nitrogen and then mechanically disrupted in a Mikro-Dismembrator (Sartorius, Gottingen, Germany) for 2 minutes at 2600 rpm. After the addition of 0.5 ml TRIzol and incubation for 10 minutes at room temperature, total RNA was isolated by acid-phenol extraction and precipitated with isopropanol, washed with 70% ethanol and dissolved in 100 µl of RNase-free water. Subsequently, RNA was DNase-treated using the RNase-Free DNase Set (Qiagen, Venlo, The Netherlands) and purified using the RNA Clean-Up and Concentration Micro Kit (Norgen, Thorold, Canada). The RNA concentration was measured using a NanoDrop spectrophotometer and the RNA quality was assessed with an Agilent 2100 Bioanalyzer (Agilent Technologies, Santa Clara, United States), and consistently found to be intact.

### Quantitative Reverse Transcriptase PCR

One µg of total RNA was transcribed into first strand cDNA using M-MLV reverse transcriptase (Invitrogen) and (dT) 12–18 (Invitrogen) oligo’s. Transcript levels were determined on the Icycler (Bio-Rad, Hercules, CA) using SYBR green supermix (Bio-Rad). Calculations were done using the comparative Ct method according to User Bulletin 2 (Applied Biosystems, Foster City, CA). Each reaction was performed in triplicate. Primers used in this study were synthesized by Biolegio (Nijmegen, Netherlands) and the sequences were as follows: GAPDH-for CATCAAGAAGGTGGTGAAGC, GAPDH-rev ACCACCCTGTTGCTGTAG, HPRT1-for GACTTGCTCGAGATGTCA, HPRT1-rev TGTAATCCAGCAGGTCAG, TNF alpha-for TCTTCTGTCTACTGAACTTCGG, TNF alpha-rev AAGATGATCTGAGTGTGAGGG, IL-1 beta-for GGCAGGCAGTATCACTCATT, IL-1 beta-rev AAGGTGCTCATGTCCTCAT, IL-6-for CCTCTCTGCAAGAGACTTCCATCCA, IL-6-rev GGCCGTGGTTGTCACCAGCA.

### Bacterial Quantification

The surface covered by bacteria was measured using the *Threshold* function of Image J, by determining the area occupied by the 488 nm (bacteria) signal. With the same function the total surface of the tissue for each image was also calculated using the 350 nm (nuclei) signal. The bacteria to tissue ratio was calculated by dividing the surface of the 488 nm signal by the total area of the tissue detected in each image. For each time point of infection, tissue sections from 3 mice were analyzed; for each mouse 4 images of each anatomical site (subarachnoid space, cerebral cortex, septum, choroid plexus) were taken using the 350, 488 and 594 nm excitation lines. The averages of each mouse were calculated for the final quantification and statistical analysis. Quantification of the amount of bacterial signal in the brain was performed at the lowest magnification (50X) images in the same manner. For each time point of infection, 6 tissue sections from 3 mice were analyzed. The averages of each mouse were calculated and this value for 3 mice per group used for the statistical analysis.

### Scoring for Co-localization

The overlap between the *S. pneumoniae* signal (488 nm green signal in 50X total magnification images) and the vascular endothelium signal (594 nm red signal in 50X total magnification images) was generated using Image J *Co-localization threshold*. In this analysis, bacteria that co-localize with the vascular endothelium appear white, while bacteria not associated with the vasculature remain green.

### Statistical Analysis

The independent student t-test of SPSS Statistics 20 (IBM) was used for all statistical analysis. The increase in mRNA levels of inflammatory cytokines between 14 hours post infection and mock was analyzed using student t-test. For the cerebral cortex and septum, the differences between 1 hour and each of the other time points (3, 8 and 14 hours) were statistically analyzed, and for the choroid plexus only between 8 and 14 hours time points. The differences in the amount of bacteria in the 50X images between the 1 hour time point and each of the other time points (3, 8 and 14 hours) were tested in the same manner as described above. The 3 averages and standard deviations corresponding to the 3 mice of each time point, including the standard deviations, were used for the analysis.

## Results

### Progression of Pneumococcal Infection: from Bacteremia to a Disease Stage Leading to Meningitis

Mice were infected intravenously with 10^7^ CFU of *S. pneumoniae* and sacrificed at fixed time points. No serious symptoms of disease were present at 1 and 3 hours, while the CFU in the blood was 10^6^ CFU/ml after 1 hour. As disease progressed, recoverable CFU in the blood increased to 10^7^ CFU/ml at 3 hours and to 10^8^ CFU/ml after 8 hours, and did not further increase at 14 hours after infection. At eight hours and even more at fourteen hours post-infection, all mice showed clear evidence of severe pneumococcal disease, which could progress towards meningitis based on previous experience [25]. Thus, our model allowed for the uniform disease progression necessary for our spatiotemporal studies. Of note, the development of the high-grade bacteremia is required for pneumococcal translocation from the bloodstream into the CNS of mice [26].

### Overview of the Endothelium of the Brain Vasculature in the Absence of Infection

**Figure 1:**
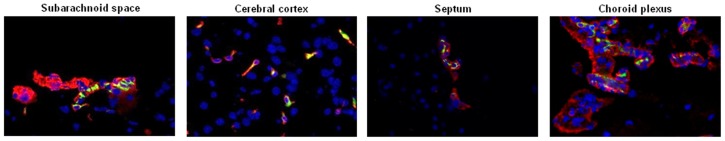
Vasculature in the different compartments of the mouse brain. Immunofluorescent detection of CD31 (488 nm green signal), tomato lectin (594 nm red signal) and nuclei (350 nm blue signal) in the brain of mock infected mice; total magnification 630X. Double staining with anti CD31 antibody and the lectin showed that the tomato lectin provides a more comprehensive detection of the brain vasculature.

The brain receives blood from two main sources, the internal carotid arteries and the vertebral arteries, which branch and narrow into the arterioles, and then branch further still into the capillaries in the inner part of the brain [27]. To visualize the brain vasculature, we used an anti CD31 antibody [28], and DyLight 594 labeled Lycopersicon Esculentum Lectin (LEL) [29], [30]. Double staining with an anti CD31 antibody and the tomato lectin showed that the latter provides a more comprehensive detection of the brain vasculature (Figure 1). Brain tissue from mock-infected mice was next used to study the vascular structures of the blood-brain barrier under uninfected conditions, as represented in Figure 1. The fluorescently labeled lectin thus enabled us to comprehensively detect the vasculature in the different mouse brain compartments and was used throughout this study.

### Spatiotemporal Distribution of S. pneumoniae in the Brain

**Figure 2:**
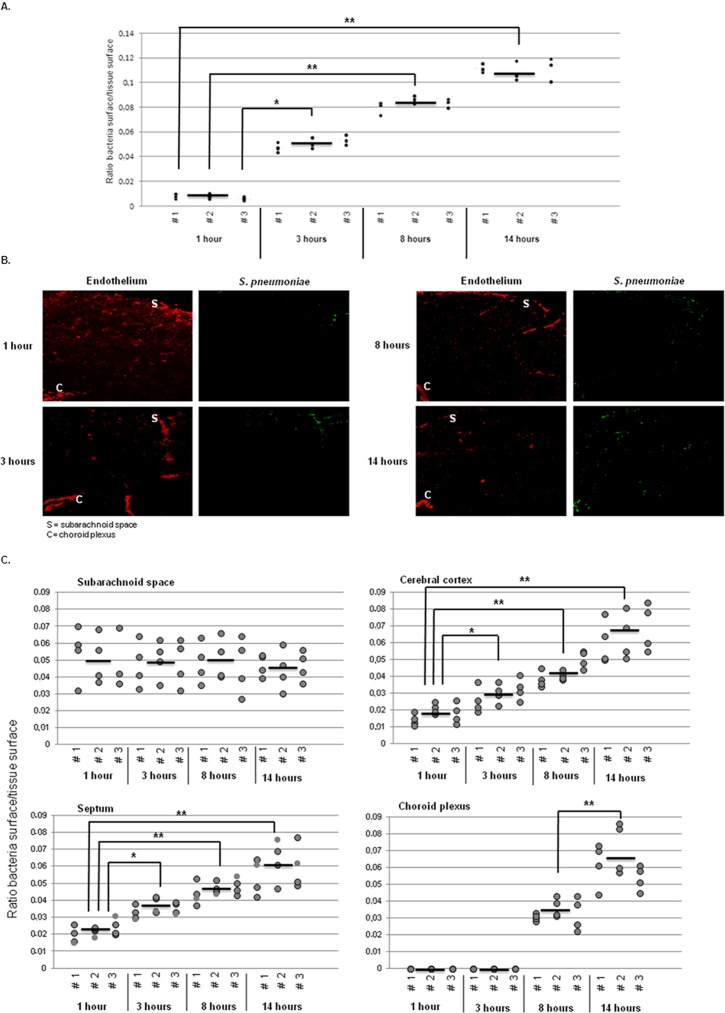
Spatiotemporal distribution of *S.* *pneumoniae* in the brain. A. Quantification of the amount of pneumococci in the brain over the time course of infection using the fluorescent signal of the secondary antibody with which the bacteria were detected. For each time point, the average signal in 9 brain sections per mouse was calculated and with the 3 averages from 3 mice, the overall average was calculated, represented as an a bold bar. In the graph, each black dot is the average value of 1 slide/mouse and each number (#1, 2, 3) represents one mouse. The ratio of bacteria in mock tissue was 0. * indicates p<0.05, ** indicates p<0.01. B. Brain slides of mice challenged with *S. pneumoniae* were stained for vasculature using tomato lectin (red) and bacteria (green) as described in Material and Methods. Total magnification 50X. These images are representative of the situation in i) each brain compartment during all the time points of infection, and ii) each mouse that was analyzed. C. Quantification of pneumococci using the fluorescent signal of the bacteria measured in the brain over the time course of infection. For each time point, the average signal in 4 brain sections per mouse was calculated and with the 3 averages from 3 mice, the overall average was calculated, represented as a black line. In the graphs, each gray dot is the value of 1 section/mouse and each number (#1, 2, 3) represents one mouse. The ratio of bacteria in mock tissue was 0. * indicates p<0.05, ** indicates p<0.01.

To measure the amount of bacteria remaining in the brain after perfusion at all time points, we applied a semi-quantitative method using the immunofluorescent signal of the specific secondary antibodies used, as described in Material and Methods. The amount of pneumococci in the brain steadily increased from 1 hour up to 14 hours after infection and difference between each time point were statistically significant (Figure 2AB). Next, we determined the amount of bacteria in the various areas of the brain using the same semi-quantification approach. As soon as 1 hour post-infection, bacteria were found in the subarachnoid vessels and over the time course of infection, the amount of bacteria detected in this site of the brain remained stable despite the 100-fold increase in bacterial titers (Figures 2C). In fact, no statistical significant differences were observed between time points in this area (Figure 2C). Bacteria were also found in the cerebral cortex and septum as soon as 1 hour after infection, and increased constantly over time in these two compartments (Figure 2C); already between 1 and 3 hours the difference was statistically significant (p value = 0.01) (Figure 2C). Remarkably, only 8 hours after infection bacteria could be detected in the choroid plexus, after which also an increase in bacterial load was observed (Figures 2A and 2C) with a statistical difference in the bacterial ratio between 8 and 14 hours (p value = 0.002).

### S. pneumoniae Adheres to the Brain Endothelium

**Figure 3:**
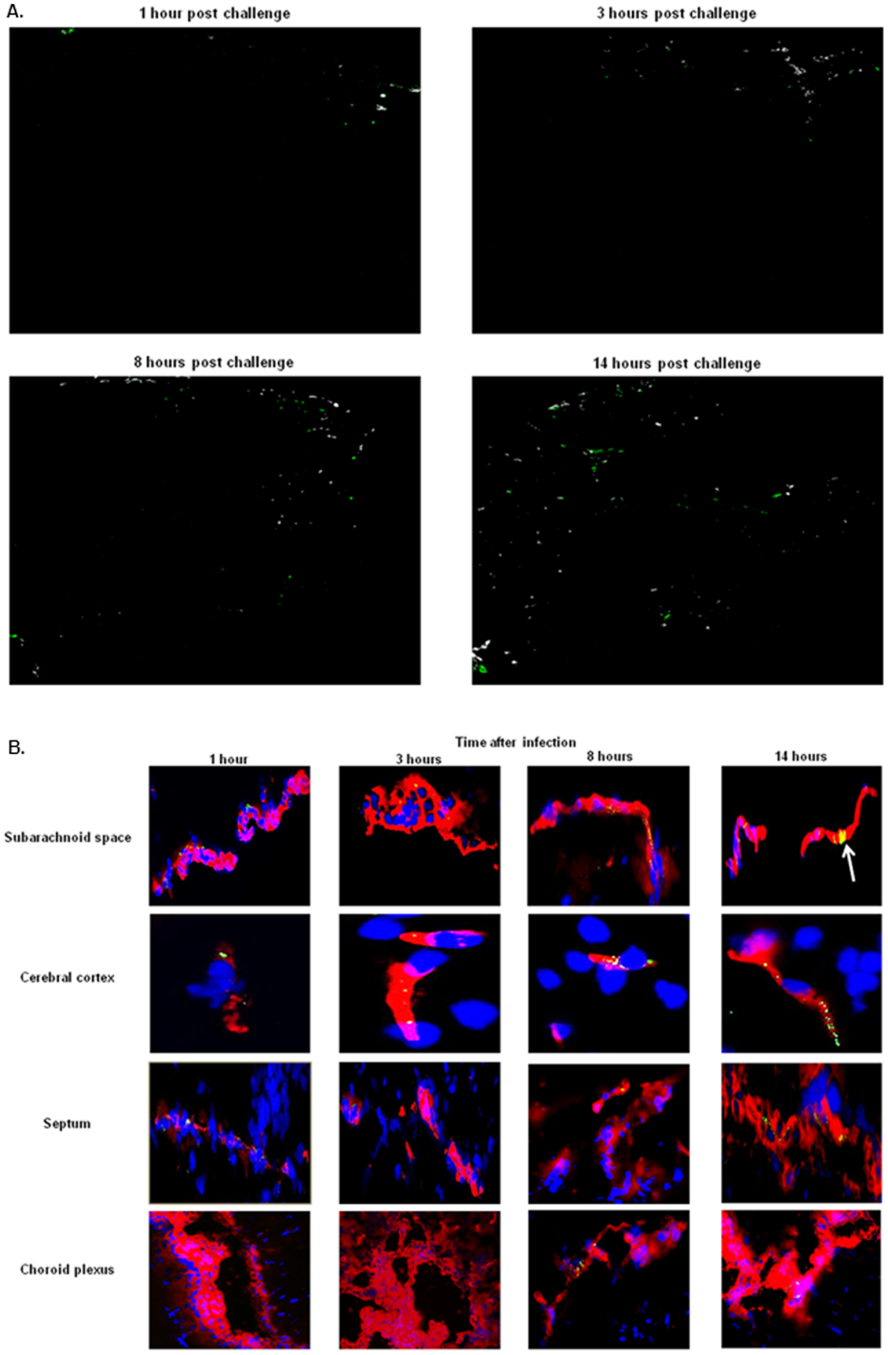
Spatiotemporal distribution of *S.* *pneumoniae* in the different compartments of the brain. A. Visualization of *S. pneumoniae* co-localized with the vasculature in the brain during the entire course of infection, same images as shown in Figure 2B; total magnification 50X. Pneumococci that co-localized with the vascular endothelium appear as white, pneumococci not co-localized appear as green. B. Brain slides of mice challenged with *S. pneumoniae* were stained for vasculature using tomato lectin (red), bacteria (green), and nuclei (blue) as described in Material and Methods. Total magnification of subarachnoid space, septum, choroid plexus 630X; total magnification of cerebral cortex 1000X. At 14 hours post infection the white arrow indicates the pneumococci forming clusters in the subarachnoid vessels. For each time point of infection, brains from 3 mice were analyzed, and of each mouse 4 brain sections were used for the immunofluorescent detection. These images are representative of the situation in i) each brain compartment during all the time course of infection and ii) each mouse that was analyzed.

Using the Co-localization Threshold function of ImageJ [24], we next determined the fraction of pneumococci which co-localized with the vascular endothelium (Figure 3A). At the earliest time points most pneumococci were associated with the blood-brain barrier endothelium. However, at the later stages of infection (8 and 14 hours time points) the amount of pneumococci whose signal did not co-localize with the brain vasculature increased (Figure 3A). Most bacteria, which were not co-localized with the blood vessels, seemed to be present in the brain tissue instead of in the lumen of the vasculature (Figure S2).

To gain further insight in pneumococcal interaction with the endothelium, detailed immune fluorescence analysis was performed on the brain slides. Pneumococci were associated with subarachnoid vessels at all time points (Figures 2B, 3A and 3B). Interestingly, in this area at the latest stage of infection (14 hours) the bacteria seemed to form clusters associated with the endothelium (Figure 3B). There were no quantitative differences between the various time points in the subarachnoid space (Figure 2C). In the cerebral cortex, only a few pneumococci were associated with the microvasculature at 1 and 3 hours post infection (Figure 3B). At the later stages of the infection, groups of *S. pneumoniae* were detected in close proximity to endothelium of the blood vessels in this area of the brain (Figure 3B). In the septum, a few bacteria were present at 1 hour time point post infection and up to 8 hours there were little changes in the amount of bacteria which co-localized with the vasculature (Figures 2C and 3B). The main difference was observed at the latest stage of infection at 14 hours, when large numbers of bacteria were observed on the endothelium of the blood vessels of this area (Figures 2C and 3B). In the choroid plexus a completely different picture emerged, in fact no *S. pneumoniae* was detected in this area of the brain during the early stages of pneumococcal infection. Only at 8 and 14 hours after infection bacteria were associated with the blood vessels (Figures 2B, 3A and 3B).

**Figure 4:**
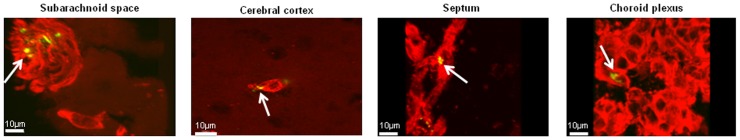
*S.* *pneumoniae* adheres to the brain vascular endothelium. Visualization of *S. pneumoniae* (green) adhering to the brain vascular endothelium (red) using confocal microscopy. The scale of each image is shown by the white scale bar. White arrows points to completely overlapping staining of the bacteria and the lectin resulting in a yellow color, strongly suggesting a co-localization of *S. pneumoniae* with endothelial cells. For each time point of infection, brains from 3 mice were analyzed, and of each mouse 3 brain sections were used for the confocal detection. The images at the later stages of infection were used because more bacteria were detected at those time points and thus these provide a clear picture of the bacteria anchored to the vasculature. Images of all time points after infection are shown in Figure S2.

To confirm that *S. pneumoniae* adhered to, or at least was in close proximity of the endothelium of the brain vessels, confocal microscopy was used. The same brain tissue slides used for the immunofluorescence analysis were studied. At all time points post- infection, brain slides were analyzed and three-dimensional reconstruction of pneumococci interacting with the brain vasculature endothelium was obtained by assembling a series of thin slices (z-stacks) taken along the vertical axis. These imaging results showed that the staining of the bacteria and the LEL signal were completely co-localized demonstrating that *S. pneumoniae* is indeed adhered tightly to the brain endothelium (Figure 4 and S2; Movies S1). In general our tissue section analysis agreed with our results from the semi-quantitative analysis and indicated that the subarachnoid space is at first the preferential site of attachment.

### Interaction of S. pneumoniae with the Blood-brain Barrier does not Cause Major Disruption of the Intercellular Junctions of Brain Endothelial Cells

**Figure 5:**
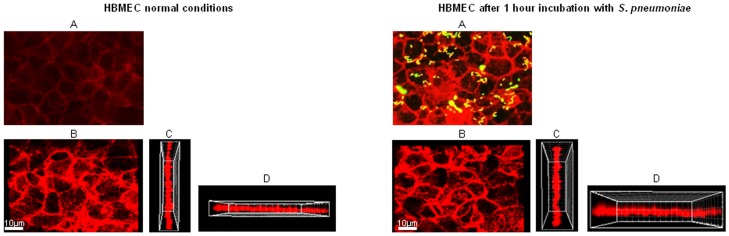
Interaction of *S.* *pneumoniae* with endothelial cells does not cause serious disruption of the intercellular junctions *in vitro*. Immunofluorescent detection of VE-Cadherin (red) and *S. pneumoniae* (green) in HBMEC in normal conditions and after 1 hour incubation with 10^6^ CFU pneumococci. Cells were stained as described in Material and Methods. Panel A shows fluorescence microscope image 630X total magnification; panel B shows confocal microscope image; panels C and D show the YZ and XZ orthogonal view planes.

The integrity of endothelium is maintained by the intercellular junctions between the endothelial cells. VE-Cadherin is an adhesion molecule expressed by the vascular endothelium and it is mainly present on the cell-cell junctions [31], [32]. For this reason this molecule was used as indicator for the loss of integrity of the endothelium [13]. VE-Cadherin staining in HBMEC after an 1 hour incubation with 10^6^ CFU of *S. pneumoniae* TIGR4 did not differ from that observed in HBMEC under normal conditions. VE-Cadherin expression was homogeneous in the intercellular junctions also in proximity of adhered bacteria, indicating no major disruptions of endothelial integrity (Figures 5A and 5B). Especially the XZ and YZ plans show that the VE-Cadherin signal is continuous without interruptions after incubation with pneumococci (Figures 5A and 5B). Furthermore, VE-Cadherin staining pattern in the subarachnoid space and choroid plexus in brain tissue of infected mice was similar to that in the brains of mock treated mice, without major signs of endothelial disruption (Figure S3).

### Leukocyte Recruitment during Pneumococcal Infection

**Figure 6:**
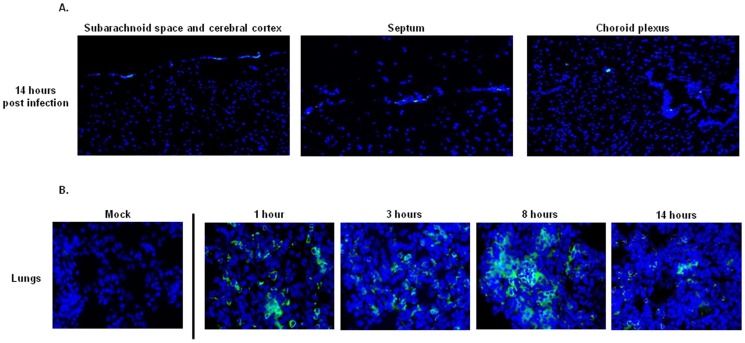
Leukocyte presence in the brain and lungs of mock treated and *S.* *pneumoniae* infected mice at different time points after infection. A. Immunofluorescent staining of the leukocyte common antigen CD45 (green) and nuclei (blue) in the subarachnoid space/cerebral cortex, septum and choroid plexus at 14 hours-post infection; total magnification 400X. The CD45 staining showed no influx of leukocytes in all the compartments of the brain in the early stages of infection (data not shown), and even 14 hours after infection the presence of leukocytes was minimal. For each time point of infection, brain sections from 3 mice were analyzed, and of each mouse 3 brain sections were used for the immunofluorescent detection. The images are representative of the situation observed in each mouse that was analyzed. B. Immunofluorescent staining of leukocyte common antigen CD45 (green) and nuclei (blue) in lungs of mock-treated and *S. pneumoniae*- infected mice; total magnification 630X. For each time point of infection, lungs from 3 mice were analyzed, and of each mouse 3 lung sections were used for the immunofluorescent detection. The images are representative of the situation observed in each mouse that was analyzed.

Inflammation is a crucial process in the defense mechanism against various infectious diseases, and leukocytes are the principal cellular mediators of inflammation. To evaluate the degree of inflammation in the brain and compare it to other tissue compartments such as the lungs, during the course of *S. pneumoniae* infection we studied tissue influx of leukocytes using the leukocyte common antigen marker CD45 [33], [34], [35]. As expected, in brain and lungs of mock treated mice no leukocytes were detected (Figure S4). The CD45 staining showed almost no influx of leukocytes in any compartment of the brain at the early stages of infection (Figure S4), and even at the later stages, the presence of leukocytes was minimal (Figure 6A). In contrast, in the lungs a different picture was observed. At all time points of pneumococcal infection, large numbers of leukocytes were present in the lungs, even as soon as after 1 hour (Figure 6B). These results reveal that intravenous injection of *S. pneumoniae* causes distinct tissue-specific responses. Furthermore, in contrast to the presence of large numbers of leukocytes in the lungs, no such infiltration was observed in the brain during the time course of our pneumococcal infection. Presumably, this would facilitate the replication of bacteria that are able to successfully translocate to the CNS.

### The Local Immune System in the Brain is Activated Early On

**Figure 7:**
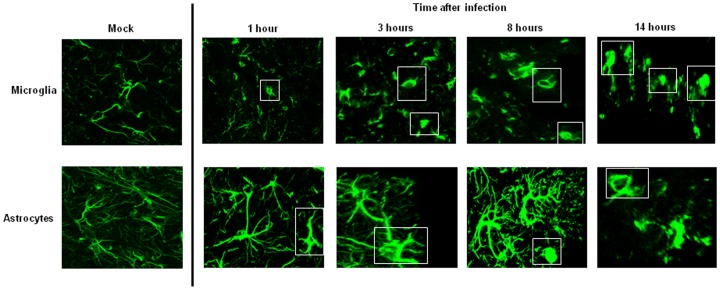
Activation of the local immune system in the brain upon pneumococcal infection. Immunofluorescent staining of Iba-1 as marker for microglia (A) and GFAP as marker for astrocytes (B) in brain of mock treated mouse and during all the time points of pneumococcal infection. Total magnification 630X. The white rectangles delineate the activated microglia and astrocytes. Brains from 3 mice for each time point were analyzed, and for each mouse 3 brain sections were used for the confocal imaging analysis. Each time point is representative the situation observed in each mouse that was analyzed.

To evaluate the local status of inflammation in the CNS during pneumococcal infection, Iba-1 [36] and GFAP [37], [38] staining was performed on mouse brain sections representative of various areas in the brain. In the mock treated tissue microglia and astrocytes had a normal cellular morphology (Figures 7, S5A and S5B). In the infected animals activated microglia and astrocytes were detected as soon as 1 hour after pneumococcal infection (Figures 7, S5A and S5B). From then on there was a gradual increase in the amount of astrogliosis and activated microglia especially at the latest time points at 8 and 14 hours, where some microglia and astrocytes had an abnormal increase in the dimensions of the soma and the dendrites were almost completely retracted (Figures 7, S5A and S5B). Thus, this analysis revealed that the local immune system in the brain is activated directly upon the presence of pneumococci in the blood.

### Inflammatory Cytokine Profile Reveals that the Infection is Progressing to Meningitis

**Figure 8:**
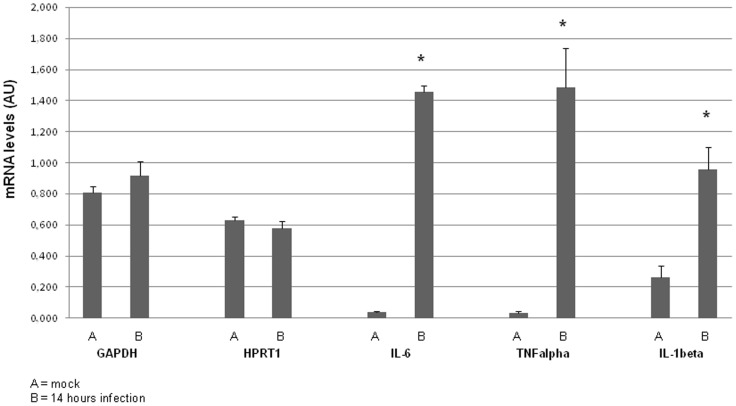
Inflammatory cytokine levels in the brain during pneumococcal infection. mRNA levels (AU = arbitrary units) of the pro-inflammatory cytokines TNF alpha, IL-1 beta, IL-6 and the housekeeping genes GAPDH and HTR1 in the brains of mock treated mice and mice sacrificed at 14 hours after infection, as quantified by Real Tim ePCR. * indicates p<0.05.

To confirm that inflammation was indeed occurring in the brain, RNA from brains of the mock treated and infected of the 14 hours time point was used to measure changes in inflammatory cytokine production. We selected IL-1 beta, IL-6 and TNF alpha for the quantitative reverse transcriptase PCR, since they are known to be the major inflammatory cytokines detected in the CSF during bacterial meningitis [39], as control the house keeping genes GAPDH and HPRT1 were used. We observed at 14 hours post infection a statistically significant 40-fold increase in levels of IL-6 and TNF alpha, and a 4-fold increase in IL-1 beta compared to the mock treated mice (Figure 8). Ct values are reported in Table S1.

## Discussion

Translocation of bacteria from the blood stream across the blood-brain barrier is hypothesized to be the principal mechanism by which the pneumococcus and other meningeal pathogens invade the CNS. A crucial moment of this translocation mechanism is the adhesion of the bacteria to the vasculature endothelium composing the blood-brain barrier. It is still unclear how and where this process takes place *in vivo.* The purpose of this study was to dissect the manner by which *S. pneumoniae* in the blood stream adheres to the endothelium of the blood-brain barrier during the events leading up to meningitis and not meningitis itself. For this reason the mice were intravenously challenged with *S. pneumoniae* and, after the sacrifice at different time points after infection, they were perfused in order to remove all bacteria not attached to the blood vessels. A second goal was to determine whether *S. pneumoniae* in the bloodstream elicits an inflammatory response from the brain before the development of meningitis.

There were several indications that pneumococcal infection in the mice was progressing towards meningitis. There was a significant, steady increase in the amount of bacteria in the brain from the first stage of infection up to 14 hours post challenge, which did not have a linear relation with the CFU’s in the blood. Our scoring method to determine which bacteria co-localized with the brain vasculature showed that at the start of infection most pneumococci were associated with the vasculature. At the later stages the amount of pneumococci not co-localized with blood vessels increased and these bacteria seemed to be present in the brain tissue. We think that these signals represent bacteria that have already crossed the blood brain barrier. This idea was strengthened by the confocal microscopy analysis, which showed that at 14 hours post infection, in the subarachnoid space, septum and choroid plexus, some pneumococci did not co-localize with the brain vasculature and seemed already in the brain (Figure S3). Additionally, at 14 hours expression of pro-inflammatory cytokines was increased significantly. On the other hand, the absence of leukocyte influx, even at 14 hours, indicates that we studied the processes leading to meningitis, but not the pathogenesis of the actual disease.

The fact that we did not observe bacteria in the choroid plexus until after 8 hours of infection in combination with the finding that the number of bacteria attached to subarachnoid vessels remained constant despite an increase in bacterial load in the blood confirmed that we did indeed remove all non-attached bacteria. The immune fluorescent analysis combined with the confocal images clearly showed that the pneumococci are indeed adhering to the brain vascular endothelium. It was hypothesized that within the subarachnoid space, bacteria grow and release proinflammatory compounds [40]. In this study we observed that at 1 hour post-infection a higher number of bacteria was observed in the subarachnoid vessels than in the other anatomical sites, implying that *S. pneumoniae* adheres preferentially to blood vessels at this site. Over time conditions seemed to change, which allowed bacterial attachment at blood vessels in distinct anatomical sites in the inner brain, such as the cerebral cortex, septum and choroid plexus. What factors are responsible for this preferential adhesion are currently unknown, but may include increased basal levels of PAF or laminin receptors. Levels of these NFkB-regulated ligands may also change during infection, explaining the increased adhesion to other sites at later time points. Future studies are warranted to determine if this is indeed the case. No increase of bacteria in the subarachnoid vessels was observed over time, which might indicate that there is no change in receptor expression at this location.

In the literature it is postulated that the choroid plexus, where the cerebrospinal fluid is produced, is the location where bacterial CNS invasion is most likely to occur [18]. Along such lines, it was shown that *S. suis* is able to pass through the blood-brain barrier at this site [19], [20]. In contrast, in our study the adherent pneumococci appeared in this area only at the later stages of infection, when levels of bacteremia were high, indicating that it is most likely not the site of initial CNS invasion. To our knowledge, this is the first study to specifically examine the spatiotemporal events of CNS invasion in the brain. Unfortunately, the level of CFUs in blood in humans that are developing pneumococcal meningitis is unknown, which makes it hard to extrapolate this data from mice with acute bacteremia to the human situation. Although events in humans with a more prolonged infection might have a distinct pathogenesis, it seems likely that the interaction of the pneumocci with the endothelium is similar.

In meningococcal meningitis *N. meningitidis* is able to form microcolonies during the passage through the blood-brain barrier [41], and we also observed *S. pneumoniae* clusters in our study. In a neonatal rat meningitis mode, breakdown of the blood-brain barrier was observed [42] and pneumolysin was thought to be the main inducer of damage to brain microvascular endothelial cells [43]. In patients with meningitis caused by *N. meningitidis* disruption of the blood-brain barrier has been shown to be a crucial event for the development of the disease [44]. In fact, during meningococcal meningitis metalloproteinases of extracellular matrix contribute to blood-brain barrier disruption [45]. Using VE-Cadherin staining, Coureuil et al. showed that *N. meningitidis* recruits the polarity complex which also plays a role in formation of tight junctions. This leads to a depletion of tight junction proteins at other places in the cell, which leads to a disruption and opening of the intercellular junctions of brain endothelial cells. After incubation of brain endothelial cells with *N. meningitidis*, openings and breaks in the intercellular junctions between endothelial cells were observed using amongst others immune fluorescent staining of VE-cadherin [13]. In our study, VE-Cadherin staining in HBMEC showed no disruption after 1 hour incubation with *S. pneumoniae* TIGR4. Furthermore, we observed a continuous and homogeneous staining of VE-Cadherin in the brains of infected mice. These findings indicate that blood-borne *S. pneumoniae* might not cause endothelium disruption during the translocation across the blood-brain barrier and support the idea of pneumococcal translocation through a pericellular or transcytosis routes [46]. Additionally, these data might indicate that toxicity of pneumolysin towards endothelial cells is limited under these conditions.

Both *in vitro* and *in vivo, S. pneumoniae* has been shown to lose its polysaccharide capsule during invasion into the host cell [47]. In our study, we primarily used antibodies that recognized the capsular polysaccharide of the bacteria. To investigate this issue in more detail, we also detected the pneumococci in the brain using a total anti-pneumococcal antiserum, recognizing capsulated and unencapsulated pneumococci, generated in a similar way as the antiserum described in Elm C. *et al*., 2004 [48]. Brains from 3 mice were analyzed for each time point and from each brain one brain section was used for the immunofluorescent detection with this serum. Interestingly, a preliminary quantification analysis indicated no differences in the amount of bacteria associated with the brain vascular endothelium compared to the data obtained with the anti-capsule antibody (data not shown). Therefore, *S. pneumoniae* might maintain its capsule during translocation over the blood-brain barrier or translocation events via an intracellular route are rare.

The inflammatory features and clinical complications following bacterial meningitis normally observed in humans can be reproduced using mouse models. Most *in vivo* models that examine the pathophysiology of bacterial meningitis consist of the direct injection of pneumococci into the brain of mice or rats [25], [49], [50]. Administration of bacteria directly into the brain bypasses the need for blood-brain barrier translocation. Our aim was to study the interaction of blood-borne *S. pneumoniae* with the brain vascular endothelium *in vivo* preceding meningitis development, hence we used a bacteremia model. We observed clear evidence of activated microglia and astrocytes already 1 hour post challenge, which implies that the local immune system in the brain is activated early on. Pneumococcal bacteremia caused a severe systemic inflammatory response, characterized by the infiltration of leukocytes in the lungs, however very little leukocyte influx in the brain was observed. Low influx of leukocytes could mean that the vascular endothelial cells are not yet activated enough over this time frame to recruit leukocytes to transmigrate over the endothelium and reach the site of infection. Mook-Kanamori et al showed neutrophil infiltration at 30 hours after infection, when meningitis is already established [25]. The inflammatory cytokine profile revealed that IL-1 beta, IL-6 and TNF alpha expression was increased at 14 hours post-injection compared to the control group. This increase was comparable to the increase observed in other studies in which the meningitis condition of the mice and rats was established by direct injection of bacteria into the cisterna magna of the brain [25], [49], [51], [52], [53]. Moreover, this increase in mRNA levels of pro-inflammatory cytokines reflects the cytokine levels measured in the CSF of hospitalized patients with bacterial meningitis that all recovered [4]. This data indicates that during bacteremia the brains displays signs of inflammation and indicates that the mice were likely progressing towards meningitis development.

The brain is able to develop an inflammatory reaction in response to lipopolysaccharides (LPS), a major component of the outer membrane of Gram-negative bacteria [54]. Previous studies by Orihuela CJ. *et al* have shown that purified pneumococcal bacterial cell wall is alone sufficient to induce apoptosis of neurons in the dentate gyrus of the hypocampus of infected mice [55]. Thus, we can at present not determine whether the adhesion of the bacteria to the endothelial cells provokes a response from the CNS or whether solely the presence of bacterial molecules in blood is the cause of the early immune activation that we observe.

In conclusion, in this study we have shown that *S. pneumoniae* preferentially adheres to the subarachnoid vessels, and over time reaches other anatomical sites in the inner brain, such as the cerebral cortex and septum. Only at the later stages of infection *S. pneumoniae* interacts with the endothelium of the choroid plexus. Furthermore, the local immune system of the brain seems to sense bacterial adhesion to the endothelial cells and is activated immediately, even before meningitis develops. Our findings suggest that *S. pneumoniae* preferentially crosses the blood brain barrier as a result of adhesion, invasion and translocation through or between endothelial cells without causing overt disruption of the vascular endothelium. This implies that *S. pneumoniae* might indeed use an intracellular or paracellular route for translocation of the blood-brain barrier. One implication of this result is that use of bacterial adhesins as vaccine candidates and or that adhesion inhibited via a pharmacological route could prevent the development of meningitis.

## Supporting Information

Figure S1 Absence of pneumococci in brain of mock treated mice. Immunofluorescent detection of tomato lectin (594 nm red signal) and nuclei (350 nm red signal) and *S. pneumoniae* (488 nm green signal) in the brain of mock treated mice; total magnification 630X. As expected, no signal for pneumococci were detected in the brain of these mice showing that the antibodies were specific for the bacteria.
(TIF)

Figure S2 Confocal microscopy visualization of *S. pneumoniae* adhered to the brain vascular endothelium. Visualization of *S. pneumoniae* (green) on the brain vascular endothelium (red) detected by confocal microscopy. Scale of each image is shown by the white scale bar, which represents 10 µm. For each time point of infection, brains from 3 mice were analyzed, and of each mouse 3 brain sections were used for the immunofluorescent detection. At 14 hours post infection the white arrow indicates the pneumococci forming clusters in the choroid plexus. These images are representative of what was observed in i) each brain compartment during all the time course of infection and ii) each mouse that was analyzed.
(TIF)

Figure S3 Integrity of intercellular junctions between endothelial cells upon *S. pneumoniae* adhesion *in vivo.* Immunofluorescent detection of VE-Cadherin (red) and *S. pneumoniae* (green) in the subarachnoid space (A) and choroid plexus (B) of mock treated and infected mice. Total magnification 630X. For each time point of infection, brains from 3 mice were analyzed, and of each mouse 3 brain sections were used for the immunofluorescent detection. The images are representative of the situation observed in each group.
(TIF)

Figure S4 Leukocyte presence in the brain of mock treated and *S. pneumoniae* infected mice at different time points after infection. Immunofluorescent staining of the leukocyte common antigen CD45 (green) and nuclei (blue) in the subarachnoid space/cerebral cortex, septum and choroid plexus in normal conditions and during the time course of pneumococcal infection; total magnification 400X. For each time point of infection, brains from 3 mice were analyzed, and of each mouse 3 brain sections were used for the immunofluorescent detection. The images are representative of the situation observed in each mouse that was analyzed.
(TIF)

Figure S5 Activation of the local immune system in the brain upon pneumococcal infection. Immunofluorescent staining of Iba-1 as marker for microglia (A) and GFAP as marker of astrocytes (B) in brain of mock treated mouse and during all the time points of pneumococcal infection. Total magnification 630X. Each number (#1, 2, 3) represents an individual mouse. Brains from 3 mice for each time point were analyzed, and for each mouse 3 brain sections were used for the confocal imaging analysis. Each time point is representative the situation observed in each mouse that was analyzed.
(TIF)

Table S1 Ct values of Quantitative Reverse Transcriptase PCR. The Ct value (cycle threshold) is defined as the number of cycles required for the fluorescent signal to cross the threshold (background level). Ct levels are inversely proportional to the amount of nucleic acids in the samples. The table shows that average of Ct values. Ct values of cytokines in mock are higher than 14 hours infection, while Ct values of house keeping genes do not vary.
(DOCX)

Movies S1 Z-stacks three-dimensional visualization of *S. pneumoniae* (green) on the brain vascular endothelium (red) detected by confocal microscopy at 8 and 14 hours after infection (z-stacks of the images shown in Figure 4). For detailed procedure see material and methods. The scale of each image is shown by the white scale bar.
(ZIP)
