# Supplementary figures and images for: Interactions between Blood-Borne Streptococcus pneumoniae and the Blood-Brain Barrier Preceding Meningitis
Source: PLoS One. 2013 Jul 16;8(7):e68408. doi: 10.1371/journal.pone.0068408 (PMC3713044; doi:10.1371/journal.pone.0068408)

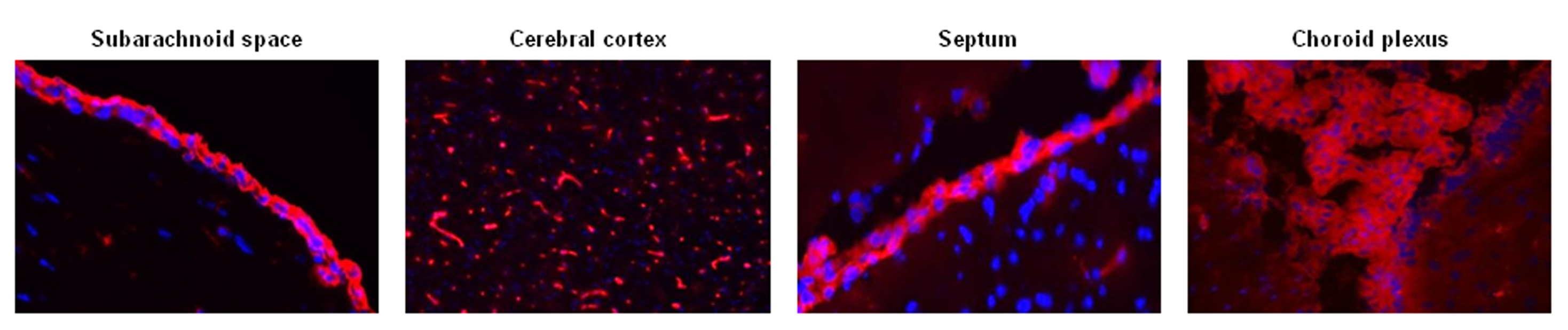

Supplement: Figure S1 — Absence of pneumococci in brain of mock treated mice. Immunofluorescent detection of tomato lectin (594 nm red signal) and nuclei (350 nm red signal) and S. pneumoniae (488 nm green signal) in the brain of mock treated mice; total magnification 630X. As expected, no signal for pneumococci were detected in the brain of these mice showing that the antibodies were specific for the bacteria. (TIF) [file pone.0068408.s001.tif]

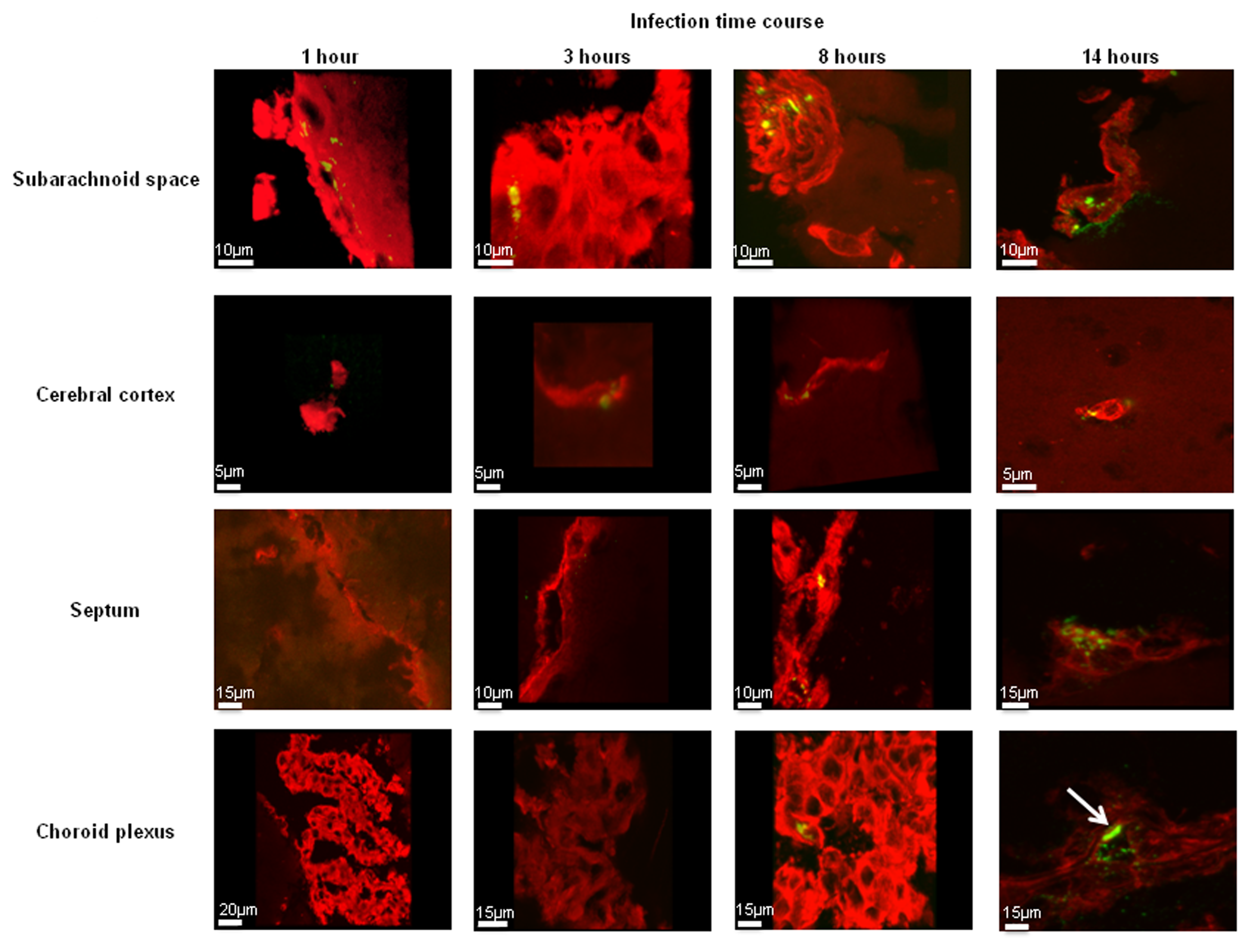

Supplement: Figure S2 — Confocal microscopy visualization of S. pneumoniae adhered to the brain vascular endothelium. Visualization of S. pneumoniae (green) on the brain vascular endothelium (red) detected by confocal microscopy. Scale of each image is shown by the white scale bar, which represents 10 µm. For each time point of infection, brains from 3 mice were analyzed, and of each mouse 3 brain sections were used for the immunofluorescent detection. At 14 hours post infection the white arrow indicates the pneumococci forming clusters in the choroid plexus. These images are representative of what was observed in i) each brain compartment during all the time course of infection and ii) each mouse that was analyzed. (TIF) [file pone.0068408.s002.tif]

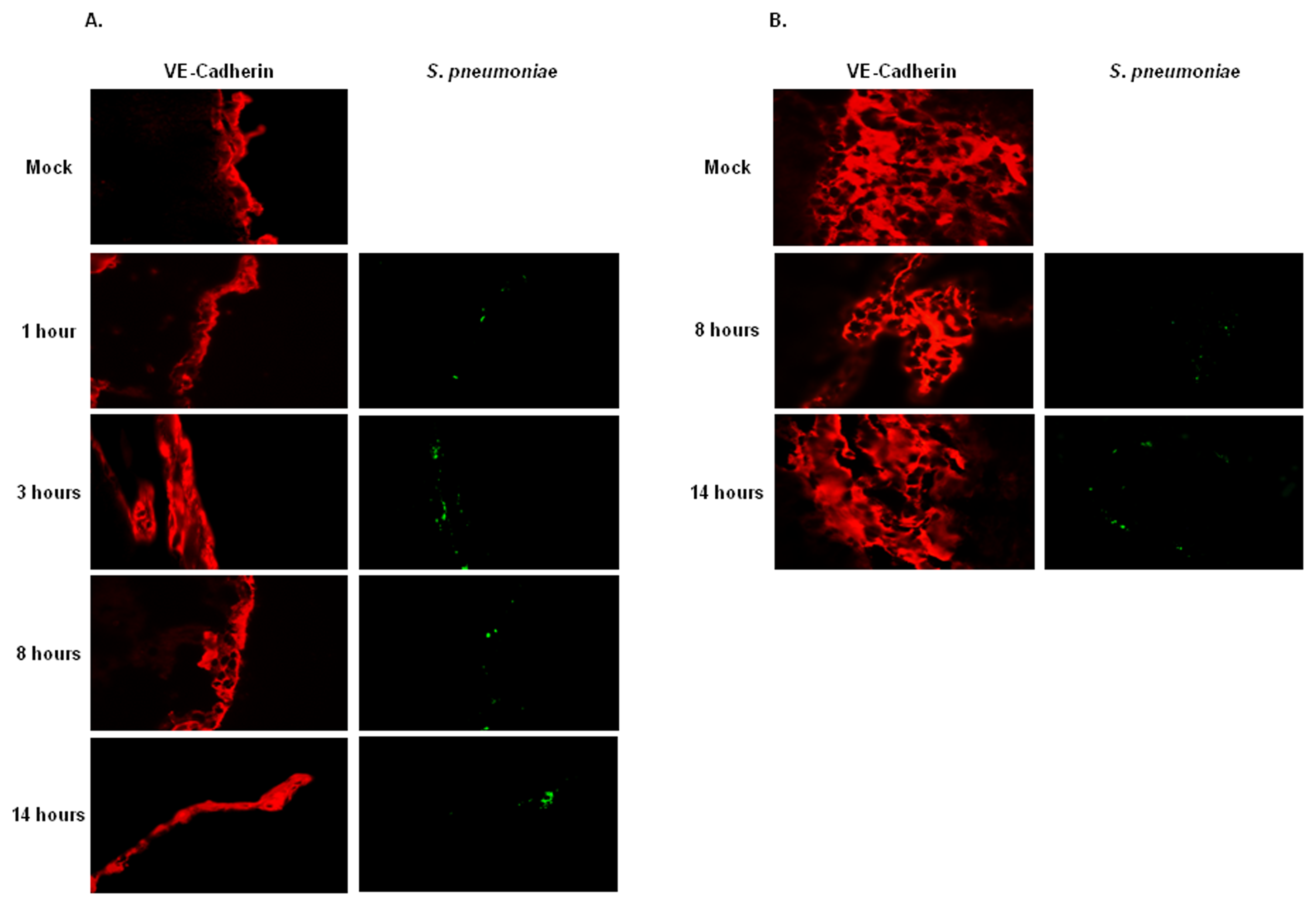

Supplement: Figure S3 — Integrity of intercellular junctions between endothelial cells upon S. pneumoniae adhesion in vivo. Immunofluorescent detection of VE-Cadherin (red) and S. pneumoniae (green) in the subarachnoid space (A) and choroid plexus (B) of mock treated and infected mice. Total magnification 630X. For each time point of infection, brains from 3 mice were analyzed, and of each mouse 3 brain sections were used for the immunofluorescent detection. The images are representative of the situation observed in each group. (TIF) [file pone.0068408.s003.tif]

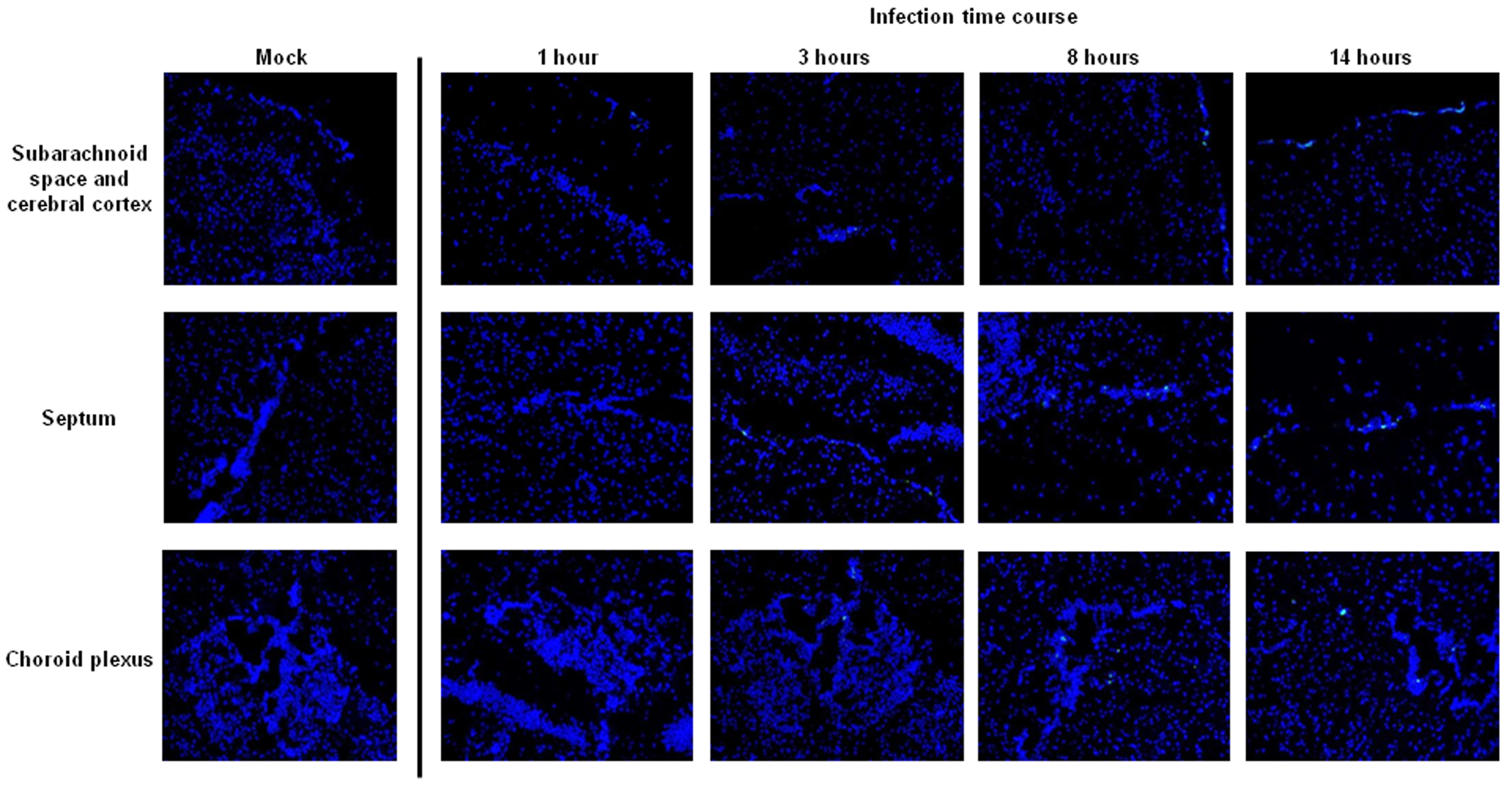

Supplement: Figure S4 — Leukocyte presence in the brain of mock treated and S. pneumoniae infected mice at different time points after infection. Immunofluorescent staining of the leukocyte common antigen CD45 (green) and nuclei (blue) in the subarachnoid space/cerebral cortex, septum and choroid plexus in normal conditions and during the time course of pneumococcal infection; total magnification 400X. For each time point of infection, brains from 3 mice were analyzed, and of each mouse 3 brain sections were used for the immunofluorescent detection. The images are representative of the situation observed in each mouse that was analyzed. (TIF) [file pone.0068408.s004.tif]

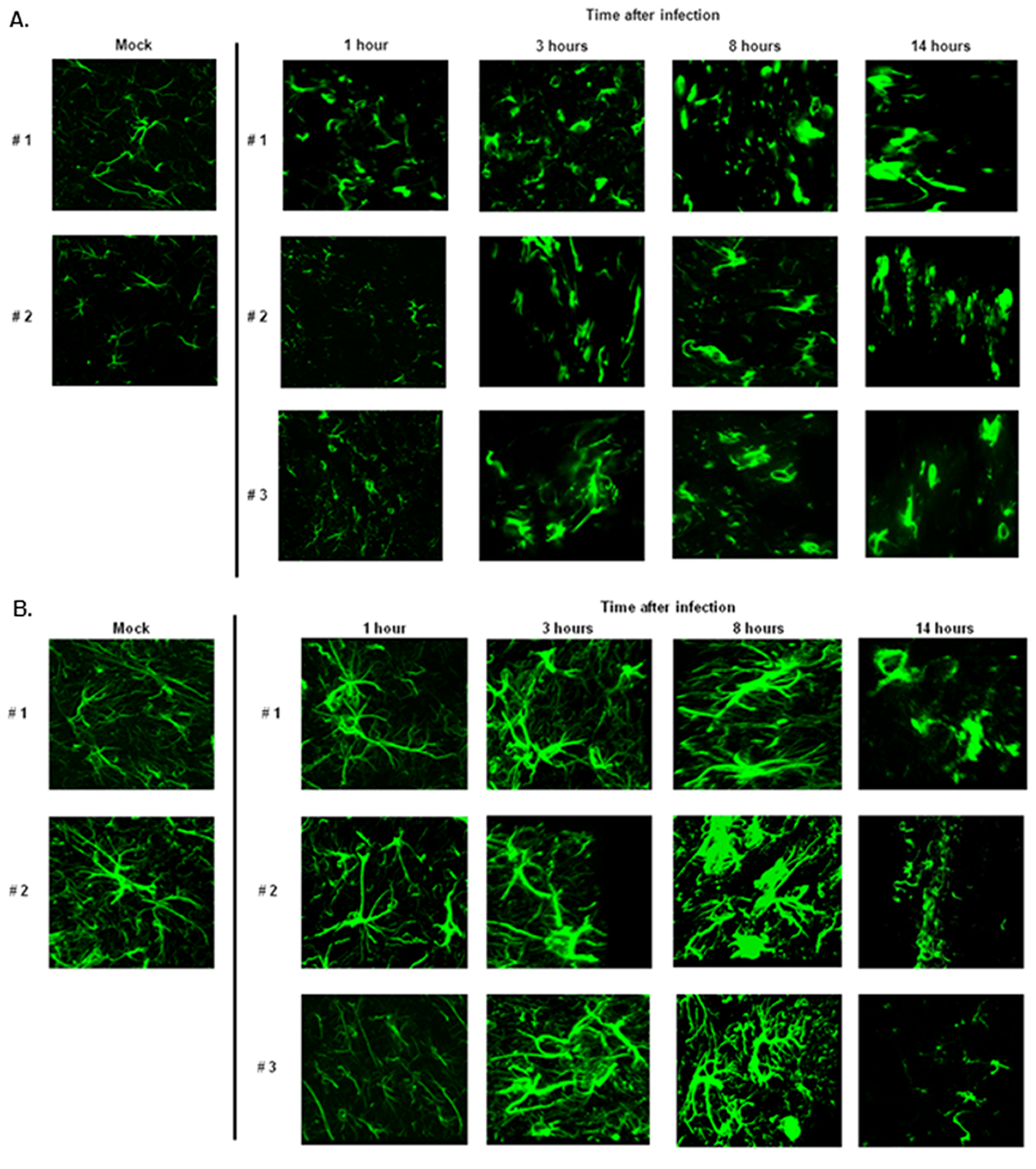

Supplement: Figure S5 — Activation of the local immune system in the brain upon pneumococcal infection. Immunofluorescent staining of Iba-1 as marker for microglia (A) and GFAP as marker of astrocytes (B) in brain of mock treated mouse and during all the time points of pneumococcal infection. Total magnification 630X. Each number (#1, 2, 3) represents an individual mouse. Brains from 3 mice for each time point were analyzed, and for each mouse 3 brain sections were used for the confocal imaging analysis. Each time point is representative the situation observed in each mouse that was analyzed. (TIF) [file pone.0068408.s005.tif]
