# Supplementary material for: Interactions between Blood-Borne Streptococcus pneumoniae and the Blood-Brain Barrier Preceding Meningitis
Source: PLoS One. 2013 Jul 16;8(7):e68408. doi: 10.1371/journal.pone.0068408 (PMC3713044; doi:10.1371/journal.pone.0068408)
Supplement: Table S1 — Ct values of Quantitative Reverse Transcriptase PCR. The Ct value (cycle threshold) is defined as the number of cycles required for the fluorescent signal to cross the threshold (background level). Ct levels are inversely proportional to the amount of nucleic acids in the samples. The table shows that average of Ct values. Ct values of cytokines in mock are higher than 14 hours infection, while Ct values of house keeping genes do not vary. (DOCX) [file pone.0068408.s006.docx]

**Table S1. Ct values of Quantitative Reverse Transcriptase PCR**

|  | **GAPDH** | **HPRT-1** | **IL-6** | **TNF alpha** | **IL-1 beta** |
| --- | --- | --- | --- | --- | --- |
| **Mock mouse 1** | 15.97 | 21.16 | 34.6 | 32.42 | 30.17 |
| **Mock mouse 2** | 16.05 | 21.12 | 34.71 | 32.48 | 30.76 |
| **14 hours mouse 1** | 15.93 | 21.18 | 28.99 | 27.46 | 28.33 |
| **14 hours mouse 2** | 15.68 | 21.29 | 29.34 | 27.52 | 28.73 |
| **14 hours mouse 3** | 15.95 | 21.40 | 29.41 | 27.53 | 28.54 |

The Ct value (cycle threshold) is defined as the number of cycles required for the fluorescent signal to cross the threshold (background level). Ct levels are inversely proportional to the amount of nucleic acids in the samples. The table shows that average of Ct values. Ct values of cytokines in mock are higher than 14 hours infection, while Ct values of house keeping genes do not vary.
